# Islamic legal perspectives on digital currencies and how they apply to Jordanian legislation

**DOI:** 10.12688/f1000research.128767.2

**Published:** 2023-08-31

**Authors:** Nasir Albalawee, Amjed S. Al Fahoum

**Affiliations:** 1 Jadara University, Irbid, Jordan; 2 Biomedical systems and Informatics Engineering Dept., Yarmouk University, Irbid, Jordan

**Keywords:** Digital Currencies, Bitcoin, Digital Representation, The Physical Presence of Currency, Functions of Money, Jordan Law.

## Abstract

**Background**: The industrial transformation requires a speedy shift to financial digitization. One of the needs for financial digitalization in the study of Islamic contracts and Islamic business law is the use of digital platforms with digital currencies. Regarding the merits and downsides of its Sharia restrictions and its halal certification, which is currently under discussion, digital currencies and perks have generated controversy in Jordan and other Islamic countries.

**Methods**: This study intends to analyze the legal foundations of digital currency from Jordanian and Islamic legal perspectives. The descriptive-qualitative research approach was utilized, and data collection processes included documentation and a literature review. All legal possibilities that may be drawn from Islamic law in order to investigate the legality of digital currencies are explored further and used to obtain the conclusions of this study.

**Results**: A review of Sharia reasons and consideration for the wellbeing of the people suggests that digital currencies in their current form are incompatible with it and must adhere to the stipulations of Islamic finance. Therefore, digital currencies are unsuitable as a store of value or wealth due to their erratic swings, lack of purchasing power, and instant responsiveness to any technical problem, technical penetration, or official circumstance. Due to market instability, digital currencies can’t be utilized to defer payments, settle debts, or repay loans.

**Conclusions**: Digital currencies are speculative; not real money. Most of those who have this money are speculators seeking a quick payoff. Sharia views digital currency trading as gambling due to its high degree of volatility. Jordan’s government should regulate digital currency use to meet demand. Digital currencies must be addressed under e-commerce laws.

## I. Introduction

The most important recent rapid development in the areas of communication, IT, banking, and business is the expansion of payment options and the rise of electronic transfer. The design of a new electronic payment system could have a high effect on privacy issues and criminal behavior related to money transfers. Therefore, a new payment system should ideally support these two conflicting objectives. ^1^ In 1983, a paper called “Blind Signatures for Untraceable Payments” was published by David Chaum, a cryptographer who came up with the idea of an encrypted payment system and started the company. ^1^ In 1990, a company called DigiCash made eCash, which was the first cryptocurrency. Therefore, cryptocurrencies existed before Bitcoin, but most people only paid little attention to them a few years after 2009. Bitcoin, the well-known digital currency, appeared in 2009 and marked the beginning of the electronic currency era. ^2^ Unlike traditional currencies, Bitcoin has no physical form, is not issued by central banks, and is not regulated by any government agency. Despite the technical and legal challenges that surround electronic currencies and the legality or illegality of dealing with them, there is no doubt that the emergence of electronic currencies has received a great deal of interest. ^2^ Even though it doesn’t fundamentally address the demands of the time, its attributes have made it a huge hit with its distributors. ^3^

Accordingly, this paper will first introduce the notion of digital currencies, before moving on to examine the legal norms governing digital currencies from an Islamic legal perspective. Electronic currencies are quite popular, as demonstrated by their widespread adoption. ^2^^,^^3^ Bitcoin is less expensive for retailers than credit cards, but these fees could increase with less storage capacity. Along with the technical difficulties, there are concerns about fraud and privacy. A cryptocurrency’s purpose is to protect its users’ privacy, but the Bitcoin identities created randomly need to be more secure. ^4^ Bitcoin was the first widespread electronic currency where money could be made and traded without a third party. Due to the popularity of digital currencies, Jordanian law must deal with its most important problems. Second, the variety of geopolitical conditions worldwide increases the demand for it. Jordanian laws linked to this study motivate the adoption of new regulations that figure out the issues with digital currencies. Also, the results and suggestions proposed for the new Jordanian law and how it will deal with Sharia would increase trust and confidence in digital currency for Jordanians and Muslims everywhere. The subject under discussion is Islamic perspectives of digital currencies, with Bitcoin serving as a prominent example. It examines the concept of cash’s digital representation and its implications for its physical presence. In addition, the discourse explores the primary functions of money in this context. Consequently, the following questions are implicit in the research problem and its objectives: What are Digital currencies? What functions and characteristics define digital currencies? What is the status of digital currencies under the law? Is there a distinct legal framework for regulating digital currencies as a result of the enactment of beneficial laws?, and What is the position of Islamic law regarding the use of digital currencies in financial transactions?

The paper will examine the issue of the Jordanian legal system of digital currencies according to Islamic jurisprudence and law in two sections addressing, respectively, digital currencies and the legal regulations governing digital currencies. The risks of using digital currencies in violation of Jordanian law and the consequences of legislative gaps in the regulation of digital currency-specific rules are discussed in this article. Furthermore, the following matters will explain how it functions, its distinctive traits, and how the law interacts with digital money, shedding light and clarifying the repercussions of “digital currencies” when correctly described. Finally, the best ways to use digital currencies from an Islamic legal perspective will be discussed, as well as whether or not a new legal framework for digital money exists as a result of current legislation.

To examine the legalization methodology of digital currency, approaches such as explaining, analyzing, and comparing are employed. First, the research problem is defined, described, and discussed. Furthermore, jurisprudence opinions and judicial jurisprudence are used to assess the legal documents that govern the subject of the inquiry. In conclusion, the comparative method is applied to determine if the digital currency can be legalized based on comparing national, Islamic, and international laws.

The paper is structured as follows: first, in the literature review, the intention to use Cryptocurrency in Jordan and definitions of digital currencies are introduced, ideas are explained, and their legal status is discussed. The methodology explains the laws regulating digital currencies by comparing the views of national legislation and Islamic jurisprudence on dealing with digital currency. The results and discussions are then extracted. Finally, conclusions and future research ideas are introduced.

## II. Literature Review

The decision to integrate cryptocurrencies into the economic framework of Jordan is a deliberate and calculated response to the dynamic and changing global financial landscape. In light of the global phenomenon of digitization in financial transactions and the emergence of blockchain technology, the government of Jordan is contemplating the integration of cryptocurrency into its financial infrastructure as a strategy to enhance modernization efforts and leverage the potential advantages associated with this novel form of digital currency. ^5^

Due to its decentralized nature, cryptographic protections, and ability to transform established financial frameworks, cryptocurrency, whose most well-known representative is Bitcoin, has drawn much attention. ^6^ A desire for novelty does not merely drive the adoption of Bitcoin in Jordan, but rather, it is motivated by an acknowledgment of the potential transformative impact it might have. ^7^ The primary driving force behind the desire to incorporate cryptocurrency stems from its capacity to augment financial inclusivity. Approximately 129,000 Jordanians, predominantly men, trade cryptocurrencies, according to a January report by the cryptocurrency payment company TripleA. ^8^ In (AlMajali et al., 2022) ^9^ presented the inaugural empirical investigation of cryptocurrency in Jordan. Consequently, their model enhances theoretical understanding by providing the initial empirical evidence within the Jordanian setting. Their initiative aims to enhance understanding of cryptocurrency by offering valuable insights and recommendations to investors, government officials, and the wider public. The first figure in their research illustrates the demographic composition of cryptocurrency users in Jordan. ^9^ In a country where certain population groups may face limited access to conventional banking services, digital currencies can function as a means to obtain financial services that surpass geographical limitations. ^10^ In addition, the prompt and efficient cross-border transactions enabled by cryptocurrencies can enhance the efficiency of remittances and international trade, potentially strengthening Jordan’s economic engagements at the international level. ^9^ In addition, adopting Bitcoin is consistent with broader worldwide patterns that emphasize the shift towards digitalization and the prevalence of cashless societies. By adopting digital currencies, Jordan can establish itself as a leader in technical progress, which might attract international investments and stimulate innovation within its territory. ^11^ Nevertheless, the endeavor necessitates a comprehensive comprehension of the complexities entailed. The utilization of cryptocurrencies in Jordan needs a thorough examination of legislative frameworks. It is imperative to balance the advantages of Bitcoin, such as expedient and cost-effective transactions, and the possible obstacles it presents, such as security risks and adherence to legal requirements. ^12^ Achieving this equilibrium will be crucial in protecting various stakeholders’ concerns, including individual users, financial institutions, and the government.

To get along with the topic, it would be imperative to distinguish between digital currencies and traditional money and their legal prospects.

The legal status of digital currencies varies across jurisdictions, with some countries recognizing them as legal tender. In contrast, others classify them as commodities or assets. For instance, digital currencies like Bitcoin are recognized as legal payment methods in Japan, subject to specific regulations. Conversely, digital currencies are not considered legal tender in countries like China, and their use is heavily restricted. Additionally, regulatory frameworks surrounding digital currencies are still evolving, with governments and financial institutions grappling with consumer protection, money laundering, and taxation issues. As a result, the legal landscape of digital currencies remains complex and subject to ongoing developments. ^13^

Bitcoin has been described as a decentralized virtual currency. Virtual currencies, such as bitcoins, are both a form of money and a payment system. However, being a decentralized system, there is no central issuer, authority, or register-keeper. ^14^ Bitcoin is unique, not because it is a virtual currency, but because it is a proof of concept for a decentralized, non-issued electronic currency. ^14^ The regulation of virtual currencies is at a very early stage. ^14^ Most regulatory systems need to be made to work well with this kind of payment system. ^14^ Nevertheless, building and keeping trust is vital to regulating new payment services and getting people to use them. ^3^^–^^14^ Most agree that proper regulation is critical for encouraging people to use new payment methods such as mobile banking and payments. ^14^ Side by side, Jordanian law still needs to develop modern laws to deal with digital currencies because they are still relatively new and depend on advanced technologies, which makes it hard for lawmakers to understand them or figure out their secrets. Also, Jordan does not have laws that directly deal with digital currencies. ^15^ The lack of laws means that a legal framework needs to be made to deal with these currencies in a way that goes within Islamic law and is in line with Jordan’s laws. This research adds to the literature on digital currency by looking at how it works and giving a clear plan for how it should be regulated. Also, it will show how Islamic legal principles can be incorporated into future or current Jordanian regulations about cryptocurrencies.

*On the otherhand, money* is a cultural norm that helps sustain economic and social systems. Various exchange systems have emerged for centuries, including barter, precious metals, fiat currency, and, most recently, a distributed digital currency based on blockchain technology. As cryptocurrencies and stablecoins have become more popular, the world’s central banks have realized that they need to provide an alternative—or let the future of money pass them—by introducing virtual money backed and issued by a central bank called central bank digital currency (CBDC). ^16^ Central banks worldwide would develop a global CBDC standard that is accepted everywhere. CBDC’s potential implications on monetary policy and liquidity, as well as its technological and economic feasibility, have been the subject of much research. ^17^^,^^18^ To do so, Omar, M. N applied the “maqsid al-shar’ah” paradigm to CBDC and uses it to explain a central Islamic principle and discuss its ethical implications. ^19^ The research results produce an ethical ecosystem that can be used to evaluate the potential of CBDC with specific characteristics and features. Such a moral environment can also be realized by basing the digital currency system’s compliance elements on the Islamic monetary framework. When used in the financial sector, technology can be used to create innovative new services, such as FinTech. There is much talk about financial technology these days. Muslim nations are fertile ground for developing Islamic banking and financial technology. ^20^ *FinTech* is an industry buzzword that uses technology to improve financial services. Due to the widespread use of mobile and smartphone technology, Islamic banking and FinTech will do well in Muslim countries. ^21^^,^^22^ However, these opportunities are currently limited because they need to be protected by a legal framework. Islamic FinTech startups struggle with regulation and Sharia FinTech research. Islamic FinTech must keep up with traditional financial developments, maintain stability, and prevent fraudulent trade. ^20^^–^^23^ For electronic transactions to grow, many groups must work together, including the central bank, public policy, international and multilateral institutions, and Islamic banking itself (through the regulatory and supervision functions).

Digital currency is defined as “currencies that do not have a tangible physical entity or physical presence and are traded via the Internet and are not subject to control or control by a central bank or because it operates outside the traditional monetary system, it is also referred to as virtual money”. ^24^ It is a digital representation of monetary value issued by entities other than the central bank of Jordan and credit institutions, and its value is generated by the voluntary acceptance of this currency. ^25^ Therefore, in Al-Bahouth, A. and later Al-Najjar, A., they characterized digital currency as a nonexistent currency that is essentially dependent on encryption. It is a decentralized currency that is not issued by a central bank, administered by any government, or tied to any local or global currency. It is electronically mined and manufactured by computers, and it is used and shared through the Internet. ^24^^,^^25^

Bitcoin, a new monetary system for electronic payment, is without a doubt the nucleus of the emergence of digital currencies. This currency is based on encryption between the two parties and is built on an anonymous system of electronic transactions, with the aim of moving away from the centralization of major banks, as they are not monitored by different types of banks and bodies and are not subject to bank laws. ^26^ The development of Bitcoin adds to our knowledge of monetary systems. The ongoing debate about how to pay for it demonstrates the value of this contribution. According to Bergstra, J.A. and Weijland, P., Bitcoin is categorized as a highly adaptable money-like informational commodity (MLIC), hence it is unnecessary to decide in advance whether or not it is a debt. By this definition, Bitcoin can start and finish its life as a non-money, with a period of “true” moneyness (as opposed to "money-likeness") in the middle. ^27^ Innovations in microfinance distribution and repayment technology for the Islamic banking model have inspired research into a commercial bank-backed initiative to bring mobile banking to Malaysia’s Islamic microfinance institutions. ^28^ From the client’s perspective, technological applications in Islamic microfinance organizations’ payment systems and repayments present challenges. ^28^

To investigate the relationship between financial inclusion and Islamic financial services in Muslim countries, Zulkhibri M., took a qualitative approach that has shown there have been improvements in the financial infrastructure of many Muslim countries over the past few decades, but this has not reached most of the population. ^29^ Only 27% of people and businesses in Muslim countries have access to formal financial services, well below the average of 51% in emerging economies. Disadvantages include money, time, distance, paperwork, distrust, and religious beliefs. ^29^ Although it represents only 0.5% of global microfinance, Islamic microfinance is small and insignificant since it does not use a cost-effective service paradigm. This research suggests that 40 million people currently excluded from the formal financial system due to their religion, can be included if Islamic wealth redistribution techniques such as awqaf, qard-al-hassan, sadaqa, and zakah are implemented. ^29^ The Islamic financial services industry has a long way to go in many Muslim countries due to its small size and inadequate infrastructure. ^30^ Crypto assets such as Bitcoin (digital money) are intriguing in Indonesia, because they have the potential to affect the global economy. Today, trade is conducted primarily via the Internet (digitalization). ^29^ There needs to be regulatory certainty for crypto investments in Indonesia. In Jubaedah *et al*., they used Islamic law and creed philosophy principles to examine crypto assets held for trading purposes in Indonesia. Information on laws, government regulations, the Fatwa DSN MUI, and the Islamic tenets of creed, witness, and shahadah is culled from scholarly library collections for the study. ^30^ This rule is only in effect for those who are open to and able to use cryptographic assets like Bitcoin. ^30^ In Indonesia as well as Jordan, it is against the law to invest in bitcoin. ^15^^,^^30^ The use of cryptocurrency as a medium of online exchange represents a novel and sustainable contribution to the growth of Indonesia’s economy. Particular guidelines are required for the use of cryptography. ^30^ Within the realm of scholarly discourse about monetary systems, it is crucial to emphasize the inherent lack of interchangeability in the functions and qualities of money. These two dimensions encompass distinct facets that, together, influence the function of money in an economy. Throughout human history, various types of currency have emerged, each with its own unique characteristics. However, despite these differences, the fundamental functions of money have remained relatively consistent. These functions include facilitating the exchange of goods and services, acting as a store of value through time, and serving as a standardized unit of measurement for financial transactions. The characteristics ascribed to money have a complex nature, frequently shaped by the level of sophistication of the cultural structure within which it functions. Within this particular setting, the examination of digital currency becomes increasingly significant as this discussion delves into the domains of both practical and inherent perspectives. The present investigation sheds light on the complex difficulties of integrating digital money into Jordan’s legal system. This endeavor necessitates thoroughly assessing its compatibility with conventional monetary functions and characteristics (Fraser & Guzmán, 2020). ^31^

Through further exploration, a comprehensive comprehension of the concurrent attributes of wealth, namely iddikhr, marghb, tadwul, miqdr, and equitability, illuminates the enigmatic intricacies of the cryptocurrency domain. Significantly, recent academic inquiries have delved into the attributes of digital currency from the perspective of Maqasid al-Shari’ah, mainly focusing on the idea of muktasab. This scholarly investigation skillfully examines the intricate relationship between the fundamental attributes of wealth and the constantly shifting landscape of cryptocurrencies. Table 1 presents a comprehensive overview of the synergies and differentiations discussed in the insights, offering a visual synthesis of the information (Akbar, 2022). ^32^ Fundamentally, recognizing the nuanced distinction between the uses and properties of money is a fundamental aspect of the ongoing discourse. The transformation of currency over time is readily apparent. However, its fundamental roles of enabling transactions, maintaining value, and establishing a uniform measuring system continue to be the foundation of its nature. In the current financial environment, the emergence of digital currency has garnered significant attention and warrants careful analysis. This examination requires a comprehensive analysis that considers both the functional alignment and the symbiotic link between inspection and the complex aspects of wealth. The current discussion concerns the potential integration of digital currency into Jordan’s legal system, highlighting the significant impact it may have on restructuring the country’s economic landscape.

There are about 1300 other cryptocurrencies on the digital currency market, such as Ethereum, Ripple, NEM, and LitCoin. On the other hand, Bitcoins are the most sought-after cryptocurrency on the market. ^33^ The fact that it cannot be traded like other electronic currencies has made investors curious about it. ^25^ In general, understanding money means understanding objects approved by the community as an intermediary tool for conducting exchanges or trade. What is meant by “approved” in this definition is that there is an agreement among community members to use one or several objects as an intermediary tool in exchange activities. Bitcoin, also known as “BTC,” is a digital currency not issued by any institutions, organizations, or governments. Bitcoin utilizes a peer-to-peer network as a distribution medium using advanced cryptographic protocols. ^34^

## III. Methodology

Understanding money’s role is essential for establishing whether or not digital currency may be considered legal tender. In forms such as paper money, money is a means of trade and a measure of the values of products and services; therefore, it has buying power. ^35^ Maurer delved into the burgeoning topic of “mobile money,” or value transfer and storage systems that are facilitated by mobile phones and are often hailed as a “signal intervention” to increase financial inclusion and bank the “unbanked” in developing countries. He discusses how economic techniques and social narratives about markets—specifically, narratives about the opportunities for profit and financial inclusion in the “payments space”—format a consumer market for mobile money and focuses on the stories that circulate in the emerging network of expertise that is calling “mobile money” into being. More importantly, he speculated on the possibility of a new form of money by asking if consumers’ usage of mobile money and airtime as currency. ^36^ In conclusion, services may be used as a kind of payment, a means of saving, and a means of protecting one’s capital.

Based on the precedent set by the Faqih (Sharia jurisprudence), it is clear that virtual currencies cannot be considered legal tender or perform the functions traditionally associated with money. ^37^ The fact that digital currency only exists in cyberspace precludes it from being utilized as payment for physical goods and services. ^38^^,^^39^ Money (also referred to as the “money supply”) is anything that is created and commonly accepted as payment for goods or debt repayment. ^40^ Economists describe money as the frequently accepted (or generally accepted) medium of exchange. ^41^ The argument that bitcoin is not money is predicated on the notion that money serves as a medium of trade, a store of value, and a unit of account. Bitcoin partially satisfies the first condition. ^42^ Money is efficient and effective if it is acceptable, divisible, homogeneous in value, durable, transportable, uncommon, and stable. ^43^ Acceptable might be interpreted to suggest that money must have intrinsic value and be desired for its own sake. Due to the lack of intrinsic value of fiat currency, acceptance standards remain difficult. The second need is that money must be easily divisible into small quantities, allowing individuals to acquire goods and services at any price. For money to be easily divided, it must be uniform or homogenous. Durability, the final condition, requires that currency be durable, not easily destroyed, and portable. “Must be scarce” signifies that money must be relatively difficult to acquire or scarce, and its value must be relatively steady through time. ^44^ Virtual currencies have their own units of account, cannot be denominated in fiat currencies, and are convertible to variable degrees. ^45^

The value of digital currencies is not an independent standard; it requires a fiat currency equivalent. Referring to Adam, cryptocurrency can still be used as a medium of exchange. ^46^ Regarding legal tender, the government declares something to be a valid form of payment, and it must be issued by a central authority. ^47^ Bitcoin is created by no commercial legal entity, limiting its use to people who make payments. ^48^ To be referred to as “money,” an asset must fulfill the following functions: (a) average payment, (b) unit of account, and (c) store of value. ^49^ Bitcoin is still a long way from supplanting fiat currency as the predominant form of legal cash. Bitcoin unit accounts are ineffective because their prices are too high for retail transactions and their splitting fees are excessive. Frequently, the bitcoin pricing of retail goods requires a significant number of left-sided zeros, making it impossible for consumers to compare prices across products and services. ^42^ Regarding the function of a store of value, the extreme price volatility of bitcoin is also a barrier to the steady storage of wealth. ^50^ In recent years, the price of Bitcoin, which can be compared to a fundamentally useless computer entry, has increased dramatically. So, cryptocurrencies are known for their high volatility, lack of buying power, and price changes in response to any technical or technological event. Moreover, they suffer from technical penetration or official legalization. Digital currencies are also unsuitable for delayed payments and cannot be used to pay off debts or loans because they are unstable and change with the market. As a result, they are unfit for use as a store of value or wealth. ^38^^,^^39^ Therefore, researchers have found that digital currencies are not the same as conventional money and cannot even replace it. This is because of the aforementioned significant variances. Additionally, digital currencies are not commonly acknowledged or utilized. As a result of the negative publicity it has received, several nations have also rejected cryptocurrencies. ^51^ For instance, the FBI closed the Silk Road case, which received considerable negative coverage. ^52^ The Central Bank of China stated in December 2013 that virtual currency has no value and is not adequately protected by law. ^51^ Cryptocurrency is not an exception to Indonesia’s monetary policy, which prohibits using any medium of exchange other than fiat money authorized for trade and payment. ^53^ Due to negative headlines, speculation, and other hazards, cryptocurrencies, and bitcoin, in particular, are viewed as investments rather than currency. ^54^ Many countries do not use cryptocurrency because its value changes quickly; some have even made it illegal to trade it in their monetary systems and with their bankers. ^55^

### III. 1. Legislative provisions governing digital currencies

As we’ve previously mentioned, the majority of Islamic countries’ laws lack legal texts that address the subject of digital currencies. ^20^^–^^22^ As a result, we will discuss the regulations that apply to digital currencies by explicating the stance of Jordanian laws on dealing with digital currencies and the position of Islamic law on digital currencies, each in accordance with its own need.

### III. 2. The status of Jordanian regulations regarding virtual currency

The Jordanian legislator is concerned about the financial policies regulating digital currencies’ economic impact. Jordan seeks to safeguard Jordanian assets against the hazards associated with digital currency. Due to a technical breakthrough or a dramatic shift in their market value, cryptocurrencies are susceptible to significant price fluctuations and the loss of their entire worth. Also, since these losses are not covered by any public or private organization known locally or globally, the person using these currencies is legally responsible for everything that happens. There will be no accountability for risk or loss. ^56^ As for national legislation, the Central Bank of Jordan responded to cryptocurrencies in Jordan beginning in 2014, when it issued its first circular prohibiting banks and all other financial institutions under its supervision, from engaging in any manner with cryptocurrencies, followed by the publication of two additional circulars in 2018 and 2019; to reinforce what was mentioned in the first circular. ^15^ As such, the attitude of the Jordanian legislature is clear and unambiguous in its refusal to deal with any digital currencies.

In accordance with Egyptian law, the Egyptian Dar Al Iftaa (the House of Islamic Legislation in Egypt) has issued a religious edict, saying that the circulation of electronic currency is unlawful in Egypt. ^57^ As was the situation in Algeria, where electronic money was forbidden under a law forbidding the acquisition, sale, use, and possession of the so-called electronic currency, and fines were levied for breaking these laws. ^57^ Though some countries, like Egypt and Algeria, have openly banned digital currencies, others, like Germany, have opened the door to trading in them. ^57^ Bitcoin is now officially accepted as legal tender in Germany. ^57^ This means that the German government must impose a tax on the profits made by companies dealing with Bitcoin, while individual transactions are exempt from taxation. Ohio, in the United States, has officially acknowledged electronic money but has imposed limits on it, such as requiring sellers to register on an official website. Additionally, New York also created the first regulatory framework for regulating electronic money operations. ^58^

### III. 3. The standpoint of Islamic law on the use of digital currency

The Islamic religion and the traditional economic system share the same vision of acceptable currency standards and their perceived economic functions. According to the two views, cryptocurrencies are illegal money even though they are an innovative way to pay for things and a tool for exchange and trading. This illegality is because they lack money’s three most important characteristics: a medium of exchange and trade, a unit of account, and a store of value. ^59^

Although these three features are present in cryptocurrencies to some extent, they are not so obvious or inherent in them as to qualify them as valid currency. In the future, these currencies can develop and become globally recognized currencies and replace traditional currencies. However, this matter takes some time and requires appropriate governance measures to organize and subject them to supervision and control to avoid the significant risks and concerns associated with dealing in this currency. ^60^

Islamic and civil laws agree that Bitcoin is an unknown, unregulated digital currency not supported by any central bank and cannot be converted into physical commodities such as gold. ^40^^–^^55^ It relies on decentralized communications and encryption to keep its data secure. ^50^ Compared to traditional currencies and online payment services like PayPal, Bitcoin has a lot of people willing to buy and sell it, and it has low transaction costs. Also, Bitcoin could grow and become a legal currency used worldwide if it is accepted by governments everywhere and is subject to the proper rules and limits. In principle, there is no legitimate objection to creating new currencies, such as digital ones or cryptocurrencies, as long as they meet the legal requirements for legal currency. ^61^

Referring to the jurisprudential judgments on the legitimacy of transacting with digital currencies, most notably Bitcoin, we discover that Islamic Sharia scholars have divergent views on the actuality of encrypted currencies. ^60^ Electronic currency must adhere to the standards and provisions outlined by Sharia law. The popularity of electronic money may also depend on how much people want it and how useful it is. Avoid using Sharia e-money for usury transactions: The exchange rate between cash and Sharia E-Money must be the same. The exchange of cash value for Sharia E-Money value must also occur in cash. Avoid spending too much money, and do not use it to buy things against Islamic law. ^62^

Buying Bitcoin, Dogecoin, and other digital currencies with fiat money is almost the same as buying currency with currency. There are two rules governing currency exchange: first, if it is the same type of item, the scale (quantity) must be the same, and payment must be given in cash. Digital money can be equated with money because it “has a price or value,” akin to gold, a valuable commodity whose price fluctuates, and the Jordanian dinar, which also has a value. However, the currency rate fluctuates as well. Then, buying and selling digital currency must follow the same rules, standards, and pillars as buying and selling physical currency. ^62^^,^^63^

To be compliant with Islamic law, a financial system must have the following requirements: It should be interest-free (Interest prohibition (IP), which implies neither interest on debt shall be requested nor paid. It should be transparent and straightforward (no misrepresentation) so as not to deceive their trading partners. Trade partners have the right to know what they’re purchasing. Additional trading partners must be free to make their own selections. The financial system should utilize legal entities. Thus, transactions using currency must involve existent commodities and services (real entities), and gambling is prohibited (GP). Lastly, Muslims are required to provide a decent portion of their income to those in need. ^64^ Bitcoin is not recognized as a form of currency since it lacks a central issuing body, a legal framework, and a supervisory body. Furthermore, there are currently no guardian-type regulations in place in Jordan. If digital currencies fall under the supervision and control of the state, then Bitcoin would be deemed a kind of money, according to the most accurate interpretation of Sharia.

## IV. Results and discussion

This study investigated the idea of digital currency as a tool for doing business in general and as a kind of electronic transaction in particular. The purpose of this study was to provide the reader with a better understanding of the notion of this currency and the opinion of Sharia and law in Jordan about its usage, as well as its characteristics and benefits.

The research results included the following elements: digital money is the consequence of technological and scientific progress, and it has evolved to keep pace with this technological era, which is the present and the future of the global economy. Electronic currency has grown extremely popular and stable among a huge number of people due to its convenience and speed in transactions, as well as its low costs because it is decentralized and does not require a significant number of formalities required by paper cash. Due to the obligation to encrypt them in order to transact with them, digital currencies offer a high level of privacy to its dealers. The majority of national laws do not establish a legal framework for digital currencies, despite their fast and massive rise. There is no legal existence for digital currencies, and the function of a currency does not apply to digital currencies; therefore, the definition of the legal status of digital currencies remains contested. Therefore, Sharia does not regard it as ordinary money that may be transacted immediately. On the other side, substantial changes in the global regime encourage the usage of digital money, primarily because individuals lack confidence in the present global financial system.

This study thoroughly examined the viability of incorporating digital currency into the legal structure of Jordan. The present investigation examined the complex terrain of digital currency adoption, comparing and contrasting the obstacles and prospects concerning conventional fiat money.

One crucial aspect of this study is distinguishing between fiat currency and crypto-commodities. Crypto-commodities refer to digital currencies that employ cryptographic techniques to ensure secure transactions, distinguishing them from fiat money, representing traditional physical cash regulated by governmental authorities. A notable distinction arises in the context of crypto-commodities, characterized by their intrinsic decentralization, which results in their lack of direct support from governmental or central authorities.

The research rigorously examined multiple variables to evaluate the viability of integrating digital currency into the legal framework of Jordan. The abovementioned elements addressed in this study include security, stability, adoption rates, and broader societal ramifications. The study sought to clarify the potential for seamless integration of digital currencies within Jordan’s economic and legal framework through a comprehensive examination of these components.

A crucial aspect of this investigation entailed thoroughly assessing the potential advantages and disadvantages associated with using digital currency. Although digital currency has the potential to enhance transaction speed and reduce expenses, it is crucial to acknowledge the associated vulnerabilities it may create, specifically concerning security issues. The report acknowledges the potential dangers associated with cybersecurity threats, hacking incidents, and fraudulent activities that may develop due to utilizing digital currencies.

Additionally, the study explored the possible ramifications of implementing digital currencies on Jordan’s economic trajectory. Proponents said the digital currency could potentially augment financial accessibility and inclusivity, particularly among marginalized people. However, these perceived advantages were carefully evaluated in light of concerns regarding the potential disruption of the established financial system.

The researchers actively and passionately delved into these complex aspects, highlighting the importance of conducting a comprehensive and nuanced assessment of the adoption of digital money in Jordan. The thorough comprehension of the complex dynamics that underlie digital currencies is crucial, as it highlights the investigation of difficulties, opportunities, and broader ramifications. Conducting a comprehensive analysis is crucial before formulating definitive judgments regarding incorporating digital currency into the legislative framework of Jordan

In summary, integrating Bitcoin in Jordan reflects the country’s desire to adopt digital transformation and foster innovation within its financial industry. The motivation behind this action stems from the aspiration to augment financial inclusivity, optimize cross-border transactions, and conform to the worldwide shift toward digitalization. Nevertheless, implementing cryptocurrency in Jordan’s economic environment necessitates careful and thorough preparation, considering the intricate factors of regulation, economics, and technology, to guarantee a seamless and prosperous integration.

## V. Conclusion

In conclusion, this paper’s detailed research sheds light on the complex landscape pertaining to the incorporation of digital currencies inside the legislative framework of Jordan. A comprehensive understanding of the contrasting characteristics of fiat currency and crypto-commodities, along with a careful assessment of the associated advantages and disadvantages, leads to a well-rounded viewpoint. Digital currencies have some advantageous characteristics, including enhanced transaction efficiency and increased financial inclusion. However, it is important to acknowledge that they also possess inherent risks, particularly in the realm of security. The fundamental differentiation between these digital entities and conventional fiat money resides in their decentralized nature, which lacks direct official support. Upon thorough examination, it becomes apparent that digital currencies, despite their potential for transformation, exhibit notable distinctions from traditional monetary systems. The implementation of utilization strategies by the Jordanian government requires a careful and thoughtful approach that takes into account various complexities, while also being in line with the country’s economic, social, and security priorities. In this paper, we examined the relationship between Islamic law and cryptocurrency legislation. To present the most accurate descriptions of digital money, a number of credible sources were analyzed to establish a correct legal definition of digital currencies, types of digital currencies, and a discussion of digital currency legal status. Furthermore, the topic of debate and comparison was the legal system and Islamic law norms related to digital money was elaborated. Therefore, this study argues, digital currencies cannot be viewed as traditional money or even take its place, due to the vast differences that have been cited, and at the same time they do not enjoy widespread acceptance and popularity, especially considering that many countries have banned the trading of digital currencies within their monetary systems.

It can be concluded in the position of Sharia on encrypted digital currencies, according to what has been documented and deduced from researchers, that the concept of currency in Sharia is not limited to gold or silver but that Sharia approves any currency that people use as pricing tool. This idea means that, in theory, there is no legal problem with making new currencies, like digital or encrypted currency, as long as they meet the Islamic legal requirements of legal currency and are subject to the same legal rules in their circulation and transactions.

From a legal point of view, a currency with a technical and immaterial value must be backed by assets with a real, tangible value or be supervised by a reputable financial institution. This requirement is to protect its dealers from fraud or considerable currency value changes.

Bitcoin’s success is due to its use as a tool for price speculation rather than as a real currency. Most of those who bought this currency are speculators who aim to make a quick profit from speculation without taking on the risk of keeping it for a long time. For them, Bitcoin is an investment opportunity, not a currency that is dealt with like other currencies. As long as this is the case, people who want to make money quickly may stop using it and start using a newer speculative tool instead. This situation is especially true now that technology has made it easier to invest in ways that are a lot like gambling. As a result, trading in cryptocurrencies involves high risk due to their high instability, which makes investing in them more like gambling, which Sharia rejects. Therefore, it is advised that the Jordanian state should capitalize on digital currencies and utilize the huge demand for them from people by developing legal texts governing their use. There is also a need to alter e-commerce legislation by introducing new legal texts governing the problem of dealing with electronic currencies.

## Data Availability

All data underlying the results are available as part of the article and no additional source data are required.
