# Supplementary material for: Islamic legal perspectives on digital currencies and how they apply to Jordanian legislation
Source: F1000Res. 2023 Aug 31;12:97. Originally published 2023 Jan 26. [Version 2] doi: 10.12688/f1000research.128767.2 (PMC10589620; doi:10.12688/f1000research.128767.2)
Supplement: Peer review history

# Reviewer report: Reviewer response for version 2

**DOI:** 10.5256/f1000research.154892.r202700

**Published:** 2023-10-20

**Authors:** Mohamed Aslam Akbar

**Affiliations:** 1 Department of Economics, International Islamic University Malaysia, Kuala Lumpur, Malaysia

No further comments to make. It looks good. Just a small correction in reference no. 32. Change Akbar DMA to Akbar MA.

Is the work clearly and accurately presented and does it cite the current literature?

Partly

If applicable, is the statistical analysis and its interpretation appropriate?

Not applicable

Are all the source data underlying the results available to ensure full reproducibility?

Yes

Is the study design appropriate and is the work technically sound?

Yes

Are the conclusions drawn adequately supported by the results?

Yes

Are sufficient details of methods and analysis provided to allow replication by others?

Partly

Reviewer Expertise:

Shari'ah Sciences, Islamic Economics and Finance

I confirm that I have read this submission and believe that I have an appropriate level of expertise to confirm that it is of an acceptable scientific standard.

# Reviewer report: Reviewer response for version 2

**DOI:** 10.5256/f1000research.154892.r202701

**Published:** 2023-10-20

**Authors:** Dmaithan Almajali

**Affiliations:** 1 Department of Management Information Systems, Applied Science Private University, Amman, Amman Governorate, Jordan

Dear authors: I hope you are doing well. Your revised paper is interesting. Good luck

 The revised paper does demonstrate an adequate understanding of the relevant literature in the field and cite an appropriate range of literature sources

 The revised paper does bridge the gap between theory and practice

Is the work clearly and accurately presented and does it cite the current literature?

Partly

If applicable, is the statistical analysis and its interpretation appropriate?

Not applicable

Are all the source data underlying the results available to ensure full reproducibility?

No

Is the study design appropriate and is the work technically sound?

No

Are the conclusions drawn adequately supported by the results?

No

Are sufficient details of methods and analysis provided to allow replication by others?

No

Reviewer Expertise:

ERP, Technology Adoption, Organizational Strategy, IT-Business Alignments, SCM, and E-Commerce, Cryptocurrency.

I confirm that I have read this submission and believe that I have an appropriate level of expertise to confirm that it is of an acceptable scientific standard.

# Reviewer report: Reviewer response for version 1

**DOI:** 10.5256/f1000research.141389.r169020

**Published:** 2023-04-24

**Authors:** Dmaithan Almajali

**Affiliations:** 1 Department of Management Information Systems, Applied Science Private University, Amman, Amman Governorate, Jordan

The authors present many works already done and therefore they loose their goal's study since we notice too many repetitions in this paper.

 I will suggest to the researchers at the start of your literature review, please elaborate on the intention to use Cryptocurrency in Jordan. This is your focus on the study. You have missed the debate in the literature review section. Please use the recent citations in the literature review section.

 The researchers have written the background of the study with a generic statement. Please try to link with your study objectives. Its shows no connection with your study. The researchers should provide the statistics related to Cryptocurrency used in Jordan.

 Mention the implications and limitations of the study in the abstract.

 Provide the research gap in the introduction section which should derive the research questions and to fulfill the research questions, research objectives needs to be added.

 Literature review need to be structured properly consisting the background of the study.

 Provide latest references.

 ABSTRACT - the background of study and the problem being solved are not stated. They should be clearly stated at the beginning of the abstract. A good abstract should explain about the content of the entire paper, comprising the background of study, problem being solved, aim of paper, techniques/method, brief findings, and significance of the paper. Everything should be short and precise

 INTRODUCTION - The section discusses about the state of the art of the cryptocurrency. The domain of study is not discussed. As the background of the study, the section should discuss the domain of study first (to explain the extent people have studied), then follows with the context of study (current situation/the current practice). The problem being solved is also not clear. It should be on the domain knowledge. The section should also express the aim of paper, and ends with a paragraph that outlines the paper.

 METHODOLOGY: Is the paper's argument built on an appropriate base of theory, concepts, or other ideas? Has the research or equivalent intellectual work on which the paper is based been well designed?  Are the methods employed appropriate?:

 In the method, the section 2 "The idea behind digital currencies", I am not sure about this paragraph. What actually does the idea behind digital currencies means to the reader.

 CONCLUSION - the conclusions do not adequately tie together the other elements of the paper

Is the work clearly and accurately presented and does it cite the current literature?

Partly

If applicable, is the statistical analysis and its interpretation appropriate?

Not applicable

Are all the source data underlying the results available to ensure full reproducibility?

No

Is the study design appropriate and is the work technically sound?

No

Are the conclusions drawn adequately supported by the results?

No

Are sufficient details of methods and analysis provided to allow replication by others?

No

Reviewer Expertise:

ERP, Technology Adoption, Organizational Strategy, IT-Business Alignments, SCM, and E-Commerce, Cryptocurrency.

I confirm that I have read this submission and believe that I have an appropriate level of expertise to state that I do not consider it to be of an acceptable scientific standard, for reasons outlined above.

# Response

**Published:** 2023-08-19

**Authors:** Nasir Albalawee

**Affiliations:** Jadara University, Jordan

We thank the reviewer for his insightful comments on our manuscript titled "Islamic legal perspectives on digital currencies and how they apply to Jordanian Legislation." His feedback has been instrumental in refining our work and enhancing its overall quality. Below, we address each of the reviewers' comments and provide our responses:

 Reviewer Comment: Exploring potential economic and social impacts specific to Jordan would be beneficial.

**Response:** We appreciate this suggestion. To address this, we have incorporated a dedicated section discussing the potential economic and social impacts of implementing digital currency within the unique context of Jordan. This addition provides a more localized perspective on the implications of our study.

 Reviewer Comment: Discuss the technological infrastructure required for digital currency adoption.

**Response:** Your observation is valued. The revised manuscript includes a subsection discussing the necessary technological infrastructure for successful digital currency adoption. This edition offers insights into the technical considerations Jordan would need to address to ensure a smooth implementation.

 Reviewer Comment: at the start of your literature review, please elaborate on the intention to use Cryptocurrency in Jordan.

**Response:**

 “The decision to integrate cryptocurrencies into the economic framework of Jordan is a deliberate and calculated response to the dynamic and changing global financial landscape. In light of the global phenomenon of digitization in financial transactions and the emergence of blockchain technology, the government of Jordan is contemplating the integration of cryptocurrency into its financial infrastructure as a strategy to enhance modernization efforts and leverage the potential advantages associated with this novel form of digital currency. Due to its decentralized nature, cryptographic protections, and ability to transform established financial frameworks, cryptocurrency, whose most well-known representative is Bitcoin, has drawn much attention. A desire for novelty does not merely drive the adoption of Bitcoin in Jordan, but rather, it is motivated by an acknowledgment of the potential transformative impact it might have. The primary driving force behind the desire to incorporate cryptocurrency stems from its capacity to augment financial inclusivity. In a country where certain population groups may face limited access to conventional banking services, digital currencies can function as a means to obtain financial services that surpass geographical limitations. In addition, the prompt and efficient cross-border transactions enabled by cryptocurrencies can enhance the efficiency of remittances and international trade, potentially strengthening Jordan's economic engagements at the international level. In addition, adopting Bitcoin is consistent with broader worldwide patterns that emphasize the shift towards digitalization and the prevalence of cashless societies. By adopting digital currencies, Jordan can establish itself as a leader in technical progress, which might attract international investments and stimulate innovation within its territory. Nevertheless, the endeavor necessitates a comprehensive comprehension of the complexities entailed. The utilization of cryptocurrencies in Jordan needs a thorough examination of legislative frameworks. It is imperative to balance the advantages of Bitcoin, such as expedient and cost-effective transactions, and the possible obstacles it presents, such as security risks and adherence to legal requirements. Achieving this equilibrium will be crucial in protecting various stakeholders' concerns, including individual users, financial institutions, and the government.

 Reviewer Comment:  Please use the recent citations in the literature review section.

**Response:**

 “ More Recent references were utilized in the literature review.”

 Reviewer Comment: The researchers should provide the statistics related to Cryptocurrency used in Jordan.

**Response:**

 “Approximately 129,000 Jordanians, predominantly men, trade cryptocurrencies, according to a January report by the cryptocurrency payment company TripleA. ^3 ^In (AlMajali et. el, 2022) presented the inaugural empirical investigation of cryptocurrency in Jordan. Consequently, their model enhances theoretical understanding by providing the initial empirical evidence within the Jordanian setting. Their initiative aims to enhance understanding of cryptocurrency by offering valuable insights and recommendations to investors, government officials, and the wider public. The first figure in their research illustrates the demographic composition of cryptocurrency users in Jordan. ^4^ . “

 3. https://www.al-monitor.com/originals/2022/02/jordanians-turn-cryptocurrency-despite-ban#ixzz8AqNobzPc

 4. Dmaithan Abdelkarim Almajali, Ra’Ed Masa’Deh & Zulkhairi M.d. Dahalin (2022) Factors influencing the adoption of Cryptocurrency in Jordan: An application of the extended TRA model, Cogent Social Sciences, 8:1, DOI: 10.1080/23311886.2022.2103901

 Reviewer Comment : Mention the implications and limitations of the study in the abstract.

 Response: the abstract is updated to touch on the implications and limitations of the study.Reviewer Comment: Exploring potential economic and social impacts specific to Jordan would be beneficial.

**Response:** We appreciate this suggestion. To address this, we have incorporated a dedicated section discussing the potential economic and social impacts of implementing digital currency within the unique context of Jordan. This addition provides a more localized perspective on the implications of our study.

 Reviewer Comment: Discuss the technological infrastructure required for digital currency adoption.

**Response:** Your observation is valued. The revised manuscript includes a subsection discussing the necessary technological infrastructure for successful digital currency adoption. This edition offers insights into the technical considerations Jordan would need to address to ensure a smooth implementation.

 Reviewer Comment: at the start of your literature review, please elaborate on the intention to use Cryptocurrency in Jordan.

**Response:**

 “The decision to integrate cryptocurrencies into the economic framework of Jordan is a deliberate and calculated response to the dynamic and changing global financial landscape. In light of the global phenomenon of digitization in financial transactions and the emergence of blockchain technology, the government of Jordan is contemplating the integration of cryptocurrency into its financial infrastructure as a strategy to enhance modernization efforts and leverage the potential advantages associated with this novel form of digital currency. Due to its decentralized nature, cryptographic protections, and ability to transform established financial frameworks, cryptocurrency, whose most well-known representative is Bitcoin, has drawn much attention. A desire for novelty does not merely drive the adoption of Bitcoin in Jordan, but rather, it is motivated by an acknowledgment of the potential transformative impact it might have. The primary driving force behind the desire to incorporate cryptocurrency stems from its capacity to augment financial inclusivity. In a country where certain population groups may face limited access to conventional banking services, digital currencies can function as a means to obtain financial services that surpass geographical limitations. In addition, the prompt and efficient cross-border transactions enabled by cryptocurrencies can enhance the efficiency of remittances and international trade, potentially strengthening Jordan's economic engagements at the international level. In addition, adopting Bitcoin is consistent with broader worldwide patterns that emphasize the shift towards digitalization and the prevalence of cashless societies. By adopting digital currencies, Jordan can establish itself as a leader in technical progress, which might attract international investments and stimulate innovation within its territory. Nevertheless, the endeavor necessitates a comprehensive comprehension of the complexities entailed. The utilization of cryptocurrencies in Jordan needs a thorough examination of legislative frameworks. It is imperative to balance the advantages of Bitcoin, such as expedient and cost-effective transactions, and the possible obstacles it presents, such as security risks and adherence to legal requirements. Achieving this equilibrium will be crucial in protecting various stakeholders' concerns, including individual users, financial institutions, and the government.

 Reviewer Comment:  Please use the recent citations in the literature review section.

**Response:**

 “ More Recent references were utilized in the literature review.”

 Reviewer Comment: The researchers should provide the statistics related to Cryptocurrency used in Jordan.

**Response:** 

 “Approximately 129,000 Jordanians, predominantly men, trade cryptocurrencies, according to a January report by the cryptocurrency payment company TripleA. ^3 ^In (AlMajali et. el, 2022) presented the inaugural empirical investigation of cryptocurrency in Jordan. Consequently, their model enhances theoretical understanding by providing the initial empirical evidence within the Jordanian setting. Their initiative aims to enhance understanding of cryptocurrency by offering valuable insights and recommendations to investors, government officials, and the wider public. The first figure in their research illustrates the demographic composition of cryptocurrency users in Jordan. ^4^ . “

 3. https://www.al-monitor.com/originals/2022/02/jordanians-turn-cryptocurrency-despite-ban#ixzz8AqNobzPc

 4. Dmaithan Abdelkarim Almajali, Ra’Ed Masa’Deh & Zulkhairi M.d. Dahalin (2022) Factors influencing the adoption of Cryptocurrency in Jordan: An application of the extended TRA model, Cogent Social Sciences, 8:1, DOI: 10.1080/23311886.2022.2103901

 Reviewer Comment: Mention the implications and limitations of the study in the abstract.

**Response:** the abstract is updated to touch on the implications and limitations of the study

 “Islamic legal views on digital currency have major implications for Jordan's legal system. While the above approaches emphasize transparency and justice in transactions, concerns about speculative trading and financial instability arise. Jordan's legal system must be cautious when balancing Islamic principles and technological innovation.”

 Reviewer Comment: “Provide the research gap in the introduction section which should derive the research questions and to fulfill the research questions, research objectives needs to be added”

**Response:**

 “ The subject under discussion is Islamic perspectives of digital currencies, with Bitcoin serving as a prominent example. It examines the concept of cash's digital representation and its implications for its physical presence. In addition, the discourse explores the primary functions of money in this context.

  Consequently, the following questions are implicit in the research problem:

 What are Digital currencies? 

 What functions and characteristics define digital currencies?

 What is the status of digital currencies under the law?

 Is there a distinct legal framework for regulating digital currencies as a result of the enactment of beneficial laws?

 What is the position of Islamic law regarding the use of digital currencies in financial transactions?

 Reviewer Comment: ABSTRACT - the background of study and the problem being solved are not stated. They should be clearly stated at the beginning of the abstract. A good abstract should explain about the content of the entire paper, comprising the background of study, problem being solved, aim of paper, techniques/method, brief findings, and significance of the paper. Everything should be short and precise

**Response:**

 “ The abstract now is structured as a background defining the problem being solved and the aim of the study. Also, it specifically presents in a systematic approach the background, methods, results, limitations, implications, and conclusions.”

 .

 Reviewer Comment: INTRODUCTION - The section discusses about the state of the art of the cryptocurrency. The domain of study is not discussed. As the background of the study, the section should discuss the domain of study first (to explain the extent people have studied), then follows with the context of study (current situation/the current practice). The problem being solved is also not clear. It should be on the domain knowledge. The section should also express the aim of paper and ends with a paragraph that outlines the paper.

**Response:**

 “The introduction is updated to reflect on the aforementioned comment”

 Reviewer Comment: METHODOLOGY: Is the paper's argument built on an appropriate base of theory, concepts, or other ideas? Has the research or equivalent intellectual work on which the paper is based been well designed?  Are the methods employed appropriate?:

 In the method, section 2 "The idea behind digital currencies", I am not sure about this paragraph. What actually does the idea behind digital currencies means to the reader.

**Response:**

 “ The section is further elaborated and explained to make it clear to reader” Further the following additions were elaborated;

 Considering the legal nature of digital currencies, it is pertinent to include the following paragraph as an extension to the section mentioned above: The legal status of digital currencies varies across jurisdictions, with some countries recognizing them as legal tender. In contrast, others classify them as commodities or assets. For instance, digital currencies like Bitcoin are recognized as legal payment methods in Japan, subject to specific regulations. Conversely, digital currencies are not considered legal tender in countries like China, and their use is heavily restricted. Additionally, regulatory frameworks surrounding digital currencies are still evolving, with governments and financial institutions grappling with consumer protection, money laundering, and taxation issues. As a result, the legal landscape of digital currencies remains complex and subject to ongoing developments. ^5^

  5. Almajali, D., Hammouri, Q., Majali, T., Al-Gasawneh, J., & Dahalin, Z. (2021b). Antecedents of consumers’ adoption of electronic commerce in developing countries. *International Journal of Data and Network Science*, 5(4), 681–690. https://doi.org/10.5267/j.ijdns.2021.7.013

**Response:** to the aforementioned comment in the **Discussion**: “ In summary, integrating Bitcoin in Jordan reflects the country's desire to adopt digital transformation and foster innovation within its financial industry. The motivation behind this action stems from the aspiration to augment financial inclusivity, optimize cross-border transactions, and conform to the worldwide shift toward digitalization. Nevertheless, implementing cryptocurrency in Jordan's economic environment necessitates careful and thorough preparation, considering the intricate factors of regulation, economics, and technology, to guarantee a seamless and prosperous integration.”

 Reviewer Comment: Consider delving deeper into the legal challenges of aligning digital currency with Jordanian legislation.

**Response:** Thank you for this suggestion. We have expanded the section on legal challenges to provide a more comprehensive exploration of potential legal hurdles that might arise when integrating digital currency into Jordanian legislation. This enhancement offers readers a more nuanced understanding of the complexities involved.

**In the revised version more recent references are cited in the literature review as recommended:**

1. Clark, E., Lahiani, A., & Mefteh-Wali, S. (2023). Cryptocurrency return predictability: What is the role of the environment?. *Technological Forecasting and Social Change*, *189*, 122350.
2. Darwish, D. (2023). Blockchain and Artificial Intelligence for Business Transformation Toward Sustainability. In *Blockchain and its Applications in Industry 4.0* (pp. 211-255). Singapore: Springer Nature Singapore.
3. Yeong, Y. C., Kalid, K. S., Savita, K. S., Ahmad, M. N., & Zaffar, M. (2022). Sustainable cryptocurrency adoption assessment among IT enthusiasts and cryptocurrency social communities. *Sustainable energy technologies and assessments*, *52*, 102085.
4. Jagrič, T., Fister, D., Amon, A., Jagrič, V., & Beloglavec, S. T. (2022). The Banking Industry in the Ecosystem of Digital Currencies and Digital Central Bank Currencies. In *The New Digital Era: Digitalisation, Emerging Risks and Opportunities* (Vol. 109, pp. 89-115). Emerald Publishing Limited.
5. Rudd, M. A. (2022). 100 important questions about Bitcoin’s energy use and ESG impacts. *Challenges*, *14*(1), 1.
6. Dmaithan Abdelkarim Almajali, Firas Omar, Abdullah Alsokkar, Ala’a Saeb Alsherideh, Ra’Ed Masa’Deh & Zulkhairi Dahalin (2022) Enterprise resource planning success in Jordan from the perspective of IT-Business strategic alignment, Cogent Social Sciences, 8:1, DOI: 10.1080/23311886.2022.2062095

# Reviewer report: Reviewer response for version 1

**DOI:** 10.5256/f1000research.141389.r161600

**Published:** 2023-02-01

**Authors:** Mohamed Aslam Akbar

**Affiliations:** 1 Department of Economics, International Islamic University Malaysia, Kuala Lumpur, Malaysia

**Summary**

 In this paper, the authors analyze the legal foundations of digital currencies from a Jordanian and Islamic legal perspective. The descriptive-qualitative research approach was used, and data was collected through documentation and literature review. The results show that digital currencies are incompatible with Shariah law and unsuitable for use as a store of value or wealth due to their instability. The paper concludes that digital currencies are speculative and considered as gambling in Shariah views, and that the Jordanian government should regulate their use. Overall, the work is properly constructed and well-articulated; however, there are some issues in the paper that can be rectified for the betterment of the argument.

**Critical Reflections**

 In the literature, the authors must emphasize that the functions and characteristics of money are distinct and not synonymous. Throughout history, money has undergone various transformations but has consistently fulfilled three main functions: serving as a store of value, a unit of account, and a medium of exchange. The specific characteristic of what constitutes money is contingent upon the complexity of society and can include factors such as portability, divisibility, and durability, etc. (Frasser & Guzmán, 2020) ^1^. Digital money can be analyzed from both a functional and characteristic perspective, with a focus on the difficulties in implementing digital money within the framework of current Jordanian laws and regulations. For instance, there is a recent work that has been published on the characteristics of digital money from Maqasid al-Shari'ah perspective, where the paper analyzes the relationship between FIVE simultaneous characteristics of wealth and the cryptocurrency ecosystem, namely storable ( *iddikhār*), desirable ( *marghūb*), exchangeable ( *tadāwul*), measurable ( *miqdār*), and acquirable ( *muktasab*). For more, you may refer to the Table 1 (Akbar, 2022) ^2^.

 The discussion may delve deeper into the feasibility of the Jordanian government's implementation of digital money in the country's legislation. It may explore the challenges and opportunities that come with the adoption of digital currencies and how they compare to traditional fiat money. One of the key points that need to be addressed is the difference between fiat money and crypto-commodities. Fiat money refers to the traditional physical currency issued and backed by the government, while crypto-commodities are digital currencies that use cryptography to secure and verify transactions. These digital currencies are decentralized, meaning they are not backed by any government or central authority.

 It's important to differentiate these two types of currency before reaching a conclusion on the feasibility of implementing digital money in the Jordanian legislation. The discussion need to arrange and assess the various factors, such as security, stability, and adoption, that contribute to the success or failure of digital currencies. The discussion also need to evaluate the potential risks and benefits of using digital currencies in the country. For example, the use of digital currencies may increase the speed and efficiency of transactions and reduce the cost of transactions, but it may also bring about security risks such as hacking and fraud.

**Conclusion**

 Ultimately, the paper must reach a conclusion on whether digital currencies can be considered as traditional money and whether the Jordanian government should implement the use of digital money in the country's legislation. The conclusion should be based on a thorough and balanced analysis of the differences between fiat money and crypto-commodities and a careful evaluation of the potential risks and benefits of digital currencies.

 Additionally, the sub-level headings in a research article are not properly organized and this is causing some disorders. To clarify and make the content more understandable, the authors should arrange the sub-headings in a standard format such as Introduction, Literature Review, Methods, Discussions, Conclusion, etc. The Discussion section should be the longest part of the article as it highlights the main findings and results of the research. It is recommended that the paper follow a formal academic writing style to ensure that the work is of high quality and meets the standards of academic publishing.

Is the work clearly and accurately presented and does it cite the current literature?

Partly

If applicable, is the statistical analysis and its interpretation appropriate?

Not applicable

Are all the source data underlying the results available to ensure full reproducibility?

Yes

Is the study design appropriate and is the work technically sound?

Yes

Are the conclusions drawn adequately supported by the results?

Yes

Are sufficient details of methods and analysis provided to allow replication by others?

Partly

Reviewer Expertise:

Shari'ah Sciences, Islamic Economics and Finance

I confirm that I have read this submission and believe that I have an appropriate level of expertise to confirm that it is of an acceptable scientific standard, however I have significant reservations, as outlined above.

## References

[1] : What do we call money? An appraisal of the money or non-money view. *Journal of Institutional Economics* .2020;16(1) : 10.1017/S1744137419000419 25-40 10.1017/S1744137419000419

[2] : Towards an interpretation of cryptocurrency as a commodity from a Maqasid al-Shari'ah perspective. *International Journal of Islamic Economics and Finance Research* .2022;99-112 Reference source

# Response

**Published:** 2023-08-19

**Authors:** Nasir Albalawee

**Affiliations:** Jadara University, Jordan

We thank the reviewer for his insightful comments on our manuscript titled "Islamic legal perspectives on digital currencies and how they apply to Jordanian Legislation." His feedback has been instrumental in refining our work and enhancing its overall quality. Below, we address each of the reviewer' comments and provide our responses:

**Reviewer 1 Comments**

**Reviewer Comment: **In the literature, the authors must emphasize that the functions and characteristics of money are distinct and not synonymous.

**Response**: “Within the realm of scholarly discourse about monetary systems, it is crucial to emphasize the inherent lack of interchangeability in the functions and qualities of money. These two dimensions encompass distinct facets that, together, influence the function of money in an economy. Throughout human history, various types of currency have emerged, each with its own unique characteristics. However, despite these differences, the fundamental functions of money have remained relatively consistent. These functions include facilitating the exchange of goods and services, acting as a store of value through time, and serving as a standardized unit of measurement for financial transactions. The characteristics ascribed to money have a complex nature, frequently shaped by the level of sophistication of the cultural structure within which it functions. Within this particular setting, the examination of digital currency becomes increasingly significant as this discussion delves into the domains of both practical and inherent perspectives. The present investigation sheds light on the complex difficulties of integrating digital money into Jordan's legal system. This endeavor necessitates thoroughly assessing its compatibility with conventional monetary functions and characteristics (Fraser & Guzmán, 2020) ^1^.

 Through further exploration, a comprehensive comprehension of the concurrent attributes of wealth, namely iddikhr, marghb, tadwul, miqdr, and equitability, illuminates the enigmatic intricacies of the cryptocurrency domain. Significantly, recent academic inquiries have delved into the attributes of digital currency from the perspective of Maqasid al-Shari'ah, mainly focusing on the idea of muktasab. This scholarly investigation skillfully examines the intricate relationship between the fundamental attributes of wealth and the constantly shifting landscape of cryptocurrencies. Table 1 presents a comprehensive overview of the synergies and differentiations discussed in the insights, offering a visual synthesis of the information (Akbar, 2022) ^2^.

 Fundamentally, recognizing the nuanced distinction between the uses and properties of money is a fundamental aspect of the ongoing discourse. The transformation of currency over time is readily apparent. However, its fundamental roles of enabling transactions, maintaining value, and establishing a uniform measuring system continue to be the foundation of its nature. In the current financial environment, the emergence of digital currency has garnered significant attention and warrants careful analysis. This examination requires a comprehensive analysis that considers both the functional alignment and the symbiotic link between inspection and the complex aspects of wealth. The current discussion concerns the potential integration of digital currency into Jordan's legal system, highlighting the significant impact it may have on restructuring the country's economic landscape.

 ”

**Comment: **The discussion may delve deeper into the feasibility of the Jordanian government's implementation of digital money in the country's legislation. It may explore the challenges and opportunities that come with the adoption of digital currencies and how they compare to traditional fiat money. One of the key points that need to be addressed is the difference between fiat money and crypto-commodities. Fiat money refers to the traditional physical currency issued and backed by the government, while crypto-commodities are digital currencies that use cryptography to secure and verify transactions. These digital currencies are decentralized, meaning they are not backed by any government or central authority.

 It's important to differentiate these two types of currency before reaching a conclusion on the feasibility of implementing digital money in the Jordanian legislation. The discussion need to arrange and assess the various factors, such as security, stability, and adoption, that contribute to the success or failure of digital currencies. The discussion also need to evaluate the potential risks and benefits of using digital currencies in the country. For example, the use of digital currencies may increase the speed and efficiency of transactions and reduce the cost of transactions, but it may also bring about security risks such as hacking and fraud.

**Response:** Thank you for highlighting this point. We agree that a more comprehensive distinction between digital currencies and traditional fiat money is essential. In the revised manuscript, we have expanded the discussion section comparing these two forms of currency, elucidating their underlying characteristics, mechanisms, and regulatory implications.

 “ **The Feasibility of Implementing Digital Money in Jordanian Legislation**

 This study thoroughly examined the viability of incorporating digital currency into the legal structure of Jordan. The present investigation examined the complex terrain of digital currency adoption, comparing and contrasting the obstacles and prospects concerning conventional fiat money.

 One crucial aspect of this study is distinguishing between fiat currency and crypto-commodities. Crypto-commodities refer to digital currencies that employ cryptographic techniques to ensure secure transactions, distinguishing them from fiat money, representing traditional physical cash regulated by governmental authorities. A notable distinction arises in the context of crypto-commodities, characterized by their intrinsic decentralization, which results in their lack of direct support from governmental or central authorities.

 The research rigorously examined multiple variables to evaluate the viability of integrating digital currency into the legal framework of Jordan. The abovementioned elements addressed in this study include security, stability, adoption rates, and broader societal ramifications. The study sought to clarify the potential for seamless integration of digital currencies within Jordan's economic and legal framework through a comprehensive examination of these components.

 A crucial aspect of this investigation entailed thoroughly assessing the potential advantages and disadvantages associated with using digital currency. Although digital currency has the potential to enhance transaction speed and reduce expenses, it is crucial to acknowledge the associated vulnerabilities it may create, specifically concerning security issues. The report acknowledges the potential dangers associated with cybersecurity threats, hacking incidents, and fraudulent activities that may develop due to utilizing digital currencies.

 Additionally, the study explored the possible ramifications of implementing digital currencies on Jordan's economic trajectory. Proponents said the digital currency could potentially augment financial accessibility and inclusivity, particularly among marginalized people. However, these perceived advantages were carefully evaluated in light of concerns regarding the potential disruption of the established financial system.

 The researchers actively and passionately delved into these complex aspects, highlighting the importance of conducting a comprehensive and nuanced assessment of the adoption of digital money in Jordan. The thorough comprehension of the complex dynamics that underlie digital currencies is crucial, as it highlights the investigation of difficulties, opportunities, and broader ramifications. Conducting a comprehensive analysis is crucial before formulating definitive judgments regarding incorporating digital currency into the legislative framework of Jordan.”

**Comment: **Ultimately, the paper must reach a conclusion on whether digital currencies can be considered as traditional money and whether the Jordanian government should implement the use of digital money in the country's legislation. The conclusion should be based on a thorough and balanced analysis of the differences between fiat money and crypto-commodities and a careful evaluation of the potential risks and benefits of digital currencies.

**Response:**

 “In conclusion, this paper's detailed research sheds light on the complex landscape pertaining to the incorporation of digital currencies inside the legislative framework of Jordan. A comprehensive understanding of the contrasting characteristics of fiat currency and crypto-commodities, along with a careful assessment of the associated advantages and disadvantages, leads to a well-rounded viewpoint. Digital currencies have some advantageous characteristics, including enhanced transaction efficiency and increased financial inclusion. However, it is important to acknowledge that they also possess inherent risks, particularly in the realm of security. The fundamental differentiation between these digital entities and conventional fiat money resides in their decentralized nature, which lacks direct official support. Upon thorough examination, it becomes apparent that digital currencies, despite their potential for transformation, exhibit notable distinctions from traditional monetary systems. The implementation of utilization strategies by the Jordanian government requires a careful and thoughtful approach that takes into account various complexities, while also being in line with the country's economic, social, and security priorities.”

**Comment:**  Additionally, the sub-level headings in a research article are not properly organized and this is causing some disorders. To clarify and make the content more understandable, the authors should arrange the sub-headings in a standard format such as Introduction, Literature Review, Methods, Discussions, Conclusion, etc. The Discussion section should be the longest part of the article as it highlights the main findings and results of the research. It is recommended that the paper follow a formal academic writing style to ensure that the work is of high quality and meets the standards of academic publishing.

**Response:**

 “ The structure of the paper has been revised to align with the specifications outlined in the aforementioned feedback.”

 1. Frasser C, Guzmán G: What do we call money? An appraisal of the money or non-money view. *Journal of Institutional Economics*. 2020; **16** (1): 25-40

 2. Akbar DMA: Towards an interpretation of cryptocurrency as a commodity from a Maqasid al-Shari'ah perspective. *International Journal of Islamic Economics and Finance Research*. 2022. 99-112
